# Artemis 123: development of a whole-head infant and young child MEG system

**DOI:** 10.3389/fnhum.2014.00099

**Published:** 2014-03-03

**Authors:** Timothy P. L. Roberts, Douglas N. Paulson, Eugene Hirschkoff, Kevin Pratt, Anthony Mascarenas, Paul Miller, Mengali Han, Jason Caffrey, Chuck Kincade, Bill Power, Rebecca Murray, Vivian Chow, Charlie Fisk, Matthew Ku, Darina Chudnovskaya, John Dell, Rachel Golembski, Peter Lam, Lisa Blaskey, Emily Kuschner, Luke Bloy, William Gaetz, J. Christopher Edgar

**Affiliations:** ^1^Department of Radiology, Lurie Family Foundations MEG Imaging Center, The Children’s Hospital of PhiladelphiaPhiladelphia, PA, USA; ^2^Tristan Technologies, Inc.,San Diego, CA, USA

**Keywords:** infant, young child, magnetoencephalography, resting-state, auditory

## Abstract

**Background:** A major motivation in designing the new infant and child magnetoencephalography (MEG) system described in this manuscript is the premise that electrophysiological signatures (resting activity and evoked responses) may serve as biomarkers of neurodevelopmental disorders, with neuronal abnormalities in conditions such as autism spectrum disorder (ASD) potentially detectable early in development. Whole-head MEG systems are generally optimized/sized for adults. Since magnetic field produced by neuronal currents decreases as a function of distance^2 ^and infants and young children have smaller head sizes (and thus increased brain-to-sensor distance), whole-head adult MEG systems do not provide optimal signal-to-noise in younger individuals. This spurred development of a whole-head infant and young child MEG system – Artemis 123.

**Methods:**In addition to describing the design of the Artemis 123, the focus of this manuscript is the use of Artemis 123 to obtain auditory evoked neuromagnetic recordings and resting-state data in young children. Data were collected from a 14-month-old female, an 18-month-old female, and a 48-month-old male. Phantom data are also provided to show localization accuracy.

**Results:**Examination of Artemis 123 auditory data showed generalizability and reproducibility, with auditory responses observed in all participants. The auditory MEG measures were also found to be manipulable, exhibiting sensitivity to tone frequency. Furthermore, there appeared to be a predictable sensitivity of evoked components to development, with latencies decreasing with age. Examination of resting-state data showed characteristic oscillatory activity. Finally, phantom data showed that dipole sources could be localized with an error less than 0.5 cm.

**Conclusions:**Artemis 123 allows efficient recording of high-quality whole-head MEG in infants four years and younger. Future work will involve examining the feasibility of obtaining somatosensory and visual recordings in similar-age children as well as obtaining recordings from younger infants. Thus, the Artemis 123 offers the promise of detecting earlier diagnostic signatures in such neurodevelopmental disorders.

## INTRODUCTION

A major motivation in the design of the new infant and child magnetoencephalography (MEG) system described in this manuscript is the premise that electrophysiological signatures (resting activity and evoked responses) may serve as biomarkers of neurodevelopmental disorders, with neuronal abnormalities in conditions such as autism spectrum disorder (ASD) potentially detectable very early in development. Opportunity for very early therapeutic interventions (behavioral and pharmacological) could be achieved via the use of diagnostic, stratification and neurobiological biomarkers, derived from resting, evoked and oscillatory neuronal measures.

Whereas adult whole-head MEG systems have been instrumental in the investigation of neural activity during development, adult MEG systems do not provide optimal, or in some cases even adequate, signal-to-noise ratio (SNR) in younger individuals (e.g., Gaetz et al., 2008). Specifically, the smaller head size of infants and young children leads to an increased distance between the sites of neuronal activity (brain) and MEG sensors. Further, conventional systems have an additional relatively large displacement of MEG sensor inside the helmet (~1.5–2 cm) and thus an additional head-surface to detection-coil distance. Ultimately, the distance between brain-source and MEG detection-coil (including brain-to-helmet and helmet-to-coil distances), coupled with the fact that the magnetic field strength produced by neuronal currents decreases as a function of the square of the distance, leads to smaller measurable signal amplitudes with conventional MEG detection hardware. The above factors greatly hinder the accurate detection, characterization, and localization of neuronal activity in young children.

Although the generators of neural activity can, in principle, be recorded from the scalp using electroencephalography (EEG) at least after the complete closure of the fontanelles (secondary to skull ossification) by approximately 18-months-old, the need to obtain accurate measures of left versus right hemisphere activity makes MEG a more attractive method, especially for auditory studies (e.g., see Edgar et al., 2003). Another advantage of MEG over EEG is decreased contamination from non-brain high frequency signals (e.g., microsaccades, muscle), especially when examining beta (12–30 Hz) and gamma (>30 Hz) activity (Muthukumaraswamy, 2013).

The 76-channel babySQUID®, developed in 2005, showed the advantage of placing the head closer to the MEG sensors in infants and children (Okada et al., 2006). In 2009, a whole-head MEG system was developed for infants and children (Johnson et al., 2009). This system, with a 53.4 cm circumference helmet accommodating >90% of 5-year-old US Caucasian boys, provided excellent recordings of left and right auditory responses in four-year-old subjects. As Johnson et al. note, the smaller MEG helmet allowed placement of the MEG sensors closer to the head-surface as well as a more symmetric placement of the head with respect to the MEG sensors, thus providing similar left and right hemisphere SNR (in larger adult-sized helmets the child’s head is able to move and thus is more likely to be asymmetrically placed with respect to the helmet sides). Several recent studies have reported findings from this 64-channel whole-head young child MEG system, now modified to 151-channels (PQ 1151R; Yokogawa/KIT, Kanazawa, Japan). Kikuchi et al. (2011) used this system to examine language lateralization in children aged 2- to 5-years-old, and Yoshimura et al. (2012) examined the development of 50 and 100 ms auditory responses in children aged 2- to 5-years-old. This same laboratory has recently begun to use this system to examine auditory processes in children with ASD (Kikuchi et al., 2013; Yoshimura et al., 2013). Findings from the above studies indicate that this is a fruitful area of research and emphasize the importance of whole-head (as opposed to unilateral, or partial coverage) MEG recordings in the investigation of bilateral responses as well as the study of hemispheric lateralization.

The above considerations spurred development of a novel whole-head infant and young child MEG system – the Artemis 123. This system, optimized for children 3 years and younger, was designed around a 50 cm helmet, where 50 cm represents the median head circumference of a 3-year-old child in the USA (Centre for Disease Control), thus allowing closer placement of the helmet to the underlying neuronal sources. The Artemis 123® hardware also incorporates a coil-in-vacuum sensor configuration as opposed to having the sensor geometry immersed in liquid helium. Sensors immersed in liquid helium require an insulated dewar helmet with the detection-coils necessarily located at a distance, typically greater than 1.5–2 cm, from the head-surface. The coil-in-vacuum configuration allows placement of the detection-coils (sensors) as close as 6 mm from the scalp, thereby mitigating the second source of source-sensor displacement (sensor to helmet surface distance), and providing a substantial increase in brain signal amplitude compared to a MEG system with the same sensor geometry immersed in liquid helium. Furthermore, a proprietary method for minimizing dimensional changes on cooling has an added benefit of greatly reducing the sensor’s susceptibility to vibration, such as that induced by patient motion while in the helmet.

As introduced above, a major motivation in the design of this new infant and child MEG system is the premise that electrophysiological signatures during brain development may serve as biomarkers of disease/disorder, with neuronal abnormalities potentially detectable very early in development. Specifically, growing evidence in school-aged children indicates that MEG-detected auditory cortex responses to simple tone (e.g., Gage et al., 2003; Roberts et al., 2010) and changing vowel stimuli (e.g., Roberts et al., 2011) may delineate children with disorders such as ASD from typically developing peers (for review see Kujala et al., 2013) and potentially serve as biomarkers for stratification in clinical trials. In addition to evoked response latency and amplitude measures, assessment of left and right superior temporal gyrus neural oscillatory activity in ASD [e.g., total power and inter-trial coherence (ITC)] also suggests ASD biomarkers (Wilson et al., 2007; Rojas et al., 2008; Edgar et al., 2013). The use of such auditory neural signatures as “early detection” biomarkers is predicated on the ability to measure these signals in pre-diagnostic groups. As autism is typically diagnosed by clinical presentation in young childhood, *earlier* diagnosis would require sensitivity to atypical brain activity in the young infant (<2–3 years of age). Hence the design of the Artemis 123 is optimized to detect brain activity from children ~6- to ~36-months-old.

Given this clinically motivated backdrop of sensitivity to left and right auditory evoked-field morphology in infancy and early childhood, the focus of this manuscript is on the use of Artemis 123 to obtain neuromagnetic auditory recording in children 14 to 48 months. Additionally, given findings that show resting-state oscillatory abnormalities in ASD (Cornew et al., 2012), resting-state data is also obtained to examine the feasibility of examining endogeneous brain rhythms (e.g., 8–12 Hz alpha rhythms). Finally, phantom data are provided to show the localization accuracy of the system.

## MATERIALS AND METHODS

### ARTEMIS123 HARDWARE AND SOFTWARE

**FIGURE 1:**
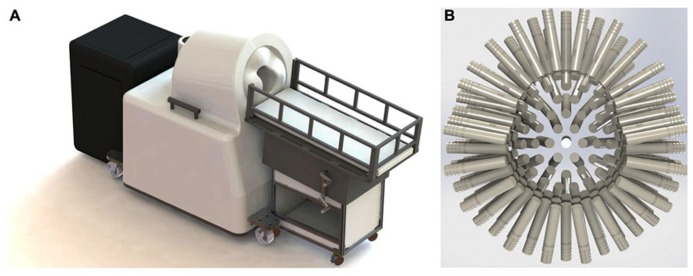
**(A)** The Tristan Technologies Inc. Artemis 123 biomagnetometer is a member of the babySQUID® product family (CAD drawing). Shown are the mobile bed, the sensor unit, and cart containing SQUID electronics, all located within the MSR. **(B)** View of the helmet and sensor array (frontal sensors on top): 123 first-order gradiometers (15 mm coil diameter and 60 mm baseline) are housed within a realistic head-shaped helmet, with whole-head coverage optimized for the median three-year-old (50 cm circumference).

The Artemis 123 biomagnetometer is a member of the babySQUID® product family, used to non-invasively measure the weak magnetic fields produced by electrical brain activity. A CAD drawing of the system is shown in **Figure 1A**. The sensors (**Figure 1B**), an array of superconducting detection-coils, are housed within a realistically shaped helmet, with whole-head coverage optimized for the median three-year-old (i.e., 50 cm circumference). The Artemis 123 sensor system is comprised of 135 channels of magnetic field pick-up coils, each connected to a low temperature SQUID (Superconducting QUantum Interference Device). The Artemis 123 is located inside a magnetic shielded room (MSR) manufactured by Vacuumschmelze (GmbH # Co. KG).

Of the 135 channels, 123 are first order axial gradiometers (number of turns in the gradiometer are +5 and -5) with a 15 mm coil diameter and 60 mm baseline. Using a coil-in-vacuum configuration, the distance from these 123 sensors to outer surface is as little as 6 mm and less than 9 mm throughout, with noise performance better than 10 fT/√Hz. The Artemis 123 also includes twelve reference channels: two sets of 3-axis magnetometers and two pairs of three reference gradiometers. Reference channels measure environmental and extraneous brain magnetic signals so that this “noise” can be removed from the brain signals of interest detected in the axial gradiometer channels.

For the Artemis 123 SQUID electronics, Tristan Technologies Inc.’s 400 series iMAG® electronics are used. These are conventional transformer-coupled SQUID electronics following a flux modulation scheme. Circuit boards are grouped in units of four channels with a local microprocessor. This architecture provides a number of important features. First, the flux modulation scheme gives the SQUIDs a flat frequency response from below 0.5 Hz to in excess of 40 kHz. Second, transformer coupling of the SQUID voltage allows the use of high-resistivity cabling in the dewar, allowing many SQUIDs to be run inside the dewar vacuum space with minimal impact on liquid helium consumption. Finally, this method allows for unshielded operation with linearity on the order of a part per million. The system sensitivity in Tesla (least-significant bit) is 0.6 fT/bit on gain 100, and the dynamic range in Tesla (least-significant bit) is +/- 250 nT on gain 1. The optimal tuning parameters for each SQUID sensor are stored in EEPROM. Although re-tuning is possible, in our experience daily tuning is generally not required as the system does not trap flux during normal operation. In addition, the Artemis 123 has no problem maintaining lock. For example, at the manufacturing facility (Tristan Technologies Inc.), it was observed that the system held lock in a completely unshielded environment, despite adjacency to a large running vacuum pump station.

**FIGURE 2:**
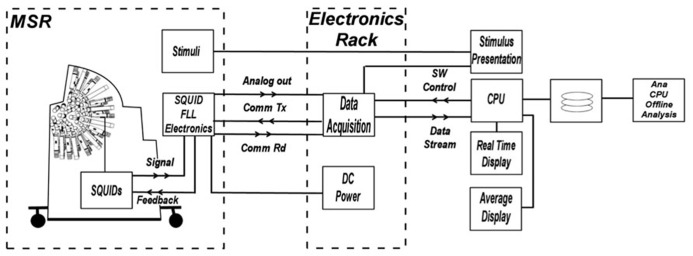
**Block diagram of electronics system functions**. The architecture provides control and real-time processing for MEG, EEG, and A/D input channels as well as fast off-line processing. The acquisition hardware consists of up to 160 simultaneously sampled 24-bit channels with built-in anti-alias filtering, plus up to an additional 96 16-bit auxiliary channels.

The Artemis 123 data acquisition system (**Figure 2**) utilizes a fiber optic linked expandable PXI architecture, enabling the acquisition chassis to reside just outside the MSR in an RF-shielded cabinet. The fiber optic link that connects the PXI chassis to the host PC running the operating software provides galvanic isolation from the electrical circuits outside the MSR while enabling data transport bandwidth of up to 80 MB/s. The acquisition hardware consists of up to 160 simultaneously sampled 24-bit channels with built-in anti-alias filtering, plus up to an additional 96 16-bit auxiliary channels. Thus, simultaneous EEG, EMG or other continuous electrical recordings are achievable. The external electronics cabinet also contains an isolation transformer and DC power supplies for the SQUID electronics in the room, and power from the supplies enter the MSR through EMI filters.

The control software for the Artemis 123 biomagnetometer runs on a PC workstation, and Windows OS. The Artemis 123 operating system is written in LabView® for rapid development and to simplify communications with National Instruments data acquisition boards. The software features simultaneous displays in a split-screen configuration (e.g., continuous data on the left and averaged data on the right). The control software is capable of graphing all channels on a strip chart display, detecting and processing event-related averages, continuously refreshing the averaged data graph, applying filters, and recording the data to disk, all at a sustained 5 kHz sample rate. The available filtering options in various points throughout the data stream, coupled with the ability to “play back” a saved data file, enables the Artemis 123 acquisition software to also be used as a post-processing tool.

**FIGURE 3:**
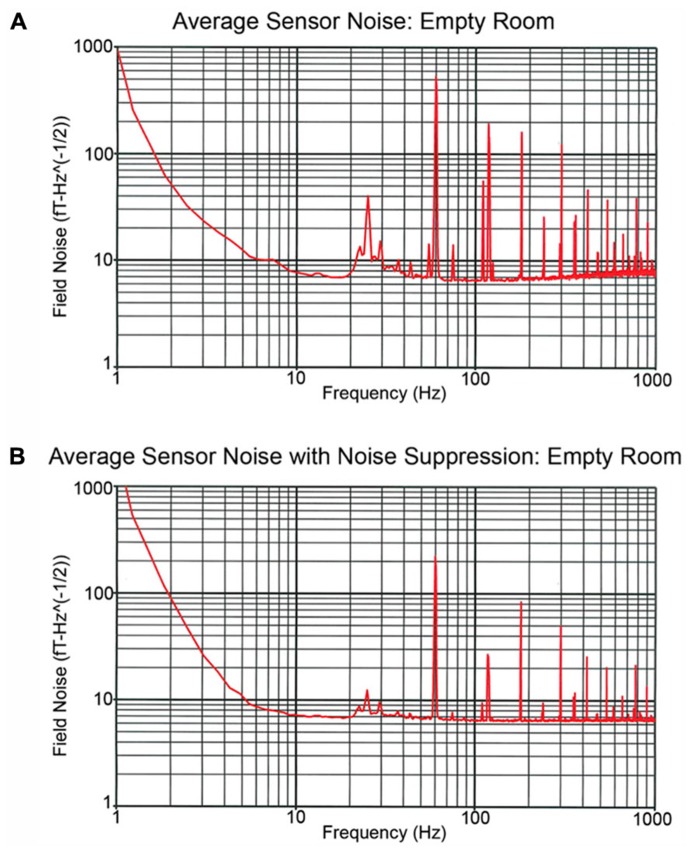
**Recordings obtained in the empty MSR**. **(A,B)** show the spectra averaged across all sensor channels. The **(A)** power spectrum shows several large peaks below 100 Hz. These include the 60 Hz powerline peak as well as a peak at ~24 Hz which reflects noise from the local mass transport/train system. In **(B)**, the reference channels were used to remove external noise, the weights obtained by calculating the covariance between signal and reference channels and then the weights applied to the raw data with no temporal filtering. **(B)** shows that via spatial filtering most of the noise activity below 100 Hz is removed.

Examining recordings obtained in the empty MSR, **Figure 3** shows two spectra, one of the raw primary sensor outputs (**Figure 3A**) and one with weights generated from the reference magnetometers applied to all sensors (**Figure 3B**) as a spatial filter to reduce external noise sources. The spectrum for each channel is the grand average of 36 averages of 8192-point FFTs (1.62 s epochs), with the figure showing the average across all sensor channels. For **Figure 3B**, prior to generation of weights, the data were band-passed from 5 to 200 Hz and then notch filters applied at the line frequency and all harmonics (i.e., 60, 120, 180, 240, and 300 Hz). Weights were obtained by calculating the covariance between signal and reference channels across the selected frequency bands. These weights were then applied to the raw data with no temporal filtering.

The **Figure 3A** power spectrum shows several large peaks below 100 Hz. These include the 60 Hz powerline peak as well as a peak at ~24 Hz which reflects noise from the local mass transport/train system. **Figure 3B** shows that via spatial filtering the reference channels can be used to remove most of the noise activity below 100 Hz. Of note, the 60 Hz peak observed in **Figure 3** is of comparable, or even smaller, amplitude than the 60 Hz peak observed in the CTF 275 system located in the same room when the CTF 3rd order noise reduction option is turned off.

### PHANTOM RECORDINGS

**FIGURE 4:**
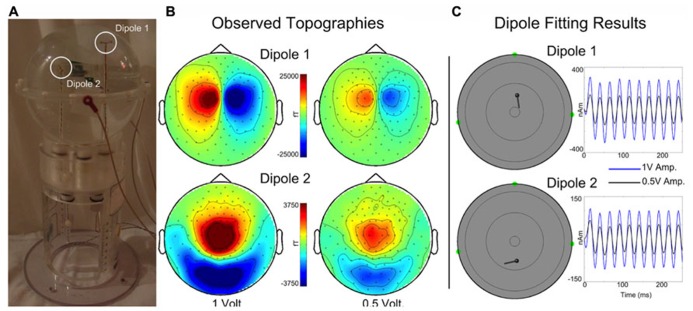
**(A)** A custom spherical saline-filled phantom with two dipole locations indicated with white circles. **(B)** Observed topographies for activated Dipoles 1 and 2 are shown for a 40 Hz sinusoidal current source at 1 and 0.5 driving voltages. **(C)** The corresponding dipole model locations and moment traces for each voltage. Estimated source localization error is less than 0.5 cm. (Of note, the Artemis 123 phantom is a scaled-down version of the CTF/MISL phantom, designed to fit into the existing CTF/MISL stand (visible in **Figure 3A**). In addition, the electrodes provided by CTF/MISL are the same as those used in the Artemis 123 phantom.).

A custom made Artemis 123 compatible spherical saline-filled electrolyte phantom was constructed (12.7 cm diameter; 40 cm circumference, see **Figure 4A**). Two current dipoles were each constructed as a pair of gold spheres of approximately 2 mm diameter with 9 mm between anode and cathode. The two current dipoles were positioned at (1.8, 0, 4.0) cm or (-1.8, 0, 2.0) cm, relative to the center of the sphere.

Dipoles were driven by a 40 Hz sinusoidal current source at each of two different driving voltages, 1 and 0.5 V. Three head position indicator coils (HPIs) were affixed to the phantom at (6.35, 0, 0) cm, (0, -6.35, 0) cm, and (0, 6.35, 0) cm to identify the phantom location within the sensor array (approximating the nasion and pre-auricular points that might be used as anatomic fiducial landmarks). The HPI coils were driven by 0.1 V sinusoidal current sources each with a unique non-harmonic frequency, 700, 750, and 800 Hz. Ten seconds of data (40 trials of 0.250 s duration) were acquired for each dipole activation and each driving voltage strength using a sample rate of 5 kHz. HPI localization was achieved by first demodulating each of the HPI signals from the sensor waveforms. Demodulated signals were then low-pass filtered (8 Hz) and averaged, yielding a single topography for each of the HPI sources. Magnetic dipoles were fit, using a combination of grid search and gradient descent algorithms, to the observed topographies, thereby identifying the locations of each of the HPIs.

For each data acquisition, a single sphere head model (radius 6.7 cm) was determined from the HPI locations. The MEG waveforms were down-sampled to 500 Hz and filtered with a 10–50 Hz band-pass. Sensor waveforms were then averaged across trials and localized by single equivalent current dipole fitting.

### HUMAN RECORDINGS

**FIGURE 5:**
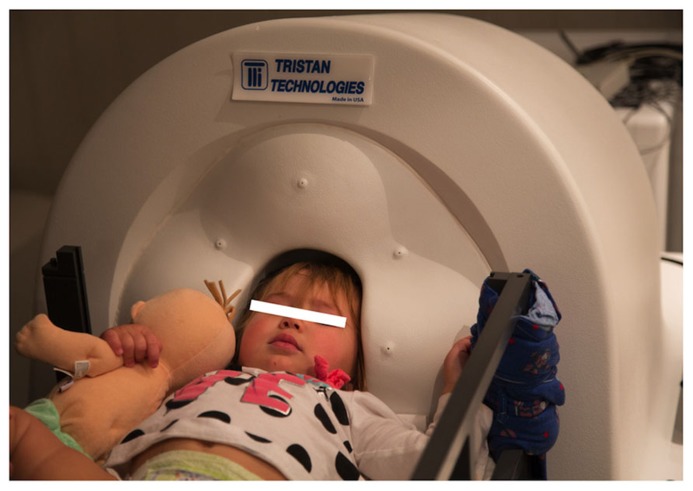
**A 14-month-old participant undergoing MEG recording**.

MEG data was collected at 5 kHz per channel. Resting-state and auditory evoked data were collected from a 14-month-old female, an 18-month-old female, and a 48-month-old male, with data obtained from all participants in a supine position (**Figure 5**). Data were obtained in the 14-month-old female while asleep, and from the two other participants while they watched a silent video projected onto the ceiling. All recordings were performed in the afternoon. All subjects were typically developing.

#### Resting-state

Resting-state data were obtained over periods of up to 60 s with minimal observed head motion. In the two awake cases, data was acquired in the eyes-open state (except for occasional blinks).

#### Auditory stimulation

500 and 1000 Hz sinusoidal tone stimuli were presented at a comfortable hearing level using a 60cm × 60cm plane-wave electrostatic directional flat-panel speaker (Panphonics SoundShower SSHP60x60, Tampere, Finland). Stimuli were of 300 ms duration (10 ms linear onset ramps) and presented with a 2 s inter-stimulus interval ( ± 0.3 s). 500 and 1000 Hz tones were presented in separate non-interleaved blocks. Stimuli were presented until at least 100 trials per condition were collected.

Auditory data were analyzed using BESA^TM^ 6.0 (BESA Gmbh, Graefelfing, Germany). Data periods exhibiting artifacts (e.g., muscle) were manually identified and removed. Artifact-free epochs were then averaged according to stimulus type and filtered using a 3 Hz (12 dB/octave, zero-phase) to 40 Hz (48 dB/octave, zero-phase) band-pass. Auditory responses were averaged over a 600 ms epoch, including a 200 ms pre-stimulus interval. The presence or absence of middle latency (~M50) and long latency (~M100) responses in the left and right hemisphere was determined by the magnetic field topography. In particular, presence of M50- and M100-like evoked response components was determined based on left and right hemisphere ingoing and outgoing flux topography (e.g., for “M100” left hemisphere ingoing anterior, outgoing posterior, and vice-versa for the right hemisphere). In view of the potential application of infant auditory MEG to detection of early signs of ASD, particular focus was placed on determining the latency of evoked responses. While latency can be determined at the sensor-level, a level of noise reduction can be obtained by principal component analysis (PCA) to more clearly delineate evoked response components. An additional SNR advantage can be achieved by estimating the neural timecourse in source space, through single equivalent dipole fitting, beamforming, or other localization techniques. In this manuscript, a simple standard brain regional source and lead field approach (with empirically optimized source orientation) to source space estimation is presented, which nonetheless confers some SNR advantage over sensor-level estimates.

Additionally, again following previous results in older children, time frequency analysis was demonstrated – using complex demodulation methods as described in Edgar et al. (2013) and implemented in BESA to yield spectrotemporal profiles of total power, evoked power and ITC from left and right hemisphere auditory sources.

## RESULTS

### PHANTOM DATA

Topographies are shown in **Figure 4B** for each dipole and drive voltage; note that for visualization purposes topologies for each dipole are scaled separately. Current dipoles were fit using the Fieldtrip software package (Oostenveld et al., 2011) to the averaged dataset, yielding locations and time courses for each dipole and drive voltage (**Figure 4C**). The mean dipole locations (each dipole was localized for 1 and 0.5 V drive amplitudes) were as follows: Dipole 1 – (1.95, -0.28, 3.93) cm, Dipole 2 – (-2.28, -0.23, 2.02) cm with standard deviations (0.03, 0.00, 0.01) cm and (0.08, 0.06, 0.16) cm. Estimated root mean square source localization error was less than 0.5 cm.

#### Resting-state

**FIGURE 6:**
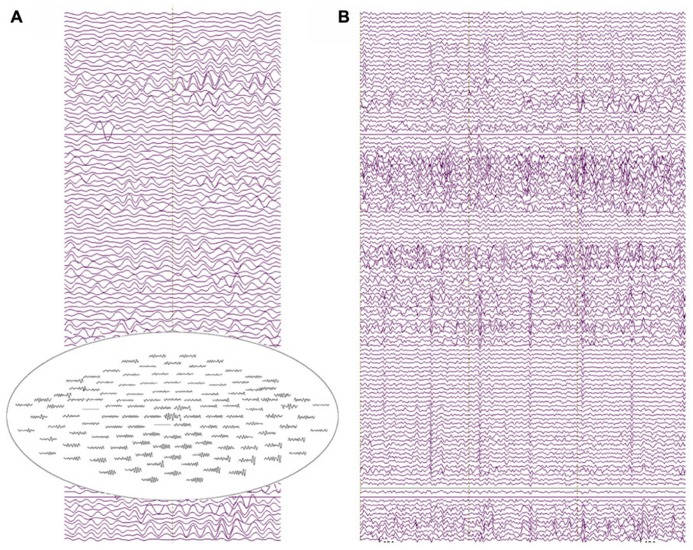
**(A)** Two seconds of resting-state recordings from an 18-month-old participant. Several channels exhibit resolvable alpha activity in the resting MEG data with increased amplitude from posterior sensor locations (see the top-view sensor inset). **(B)** 3 s of resting-state data showing heart-beat activity in most MEG channels for the same participant (approximately two heartbeats per second).

Resting-state data were obtained from all three subjects. **Figure 6** shows resting-state activity from a typical subject.

The raw recordings in the left panel show “alpha” activity (8 Hz), with the top-view inset showing the topography distinctive to resting-state alpha with alpha activity larger in occipital than frontal sensors. The panel to the right shows heartbeat in many of the channels, characteristic of clinical MEG recordings and illustrating a potential confounding artifact (note, the physical distance from heart to helmet is closer in infants than in older children or adults). Multiple “cardiac artifact” elimination schemes have been proposed (e.g., Breuer et al., 2013).

### AUDITORY DATA

**FIGURE 7:**
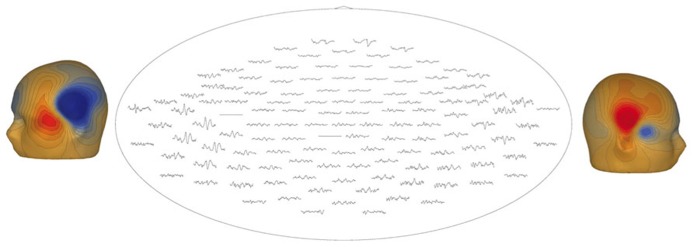
**Top-down view of MEG auditory evoked waveforms and left- and right-hemisphere “M50” magnetic field topographies for an 18-month-old participant (500 Hz tone).** In the top-down view frontal sensors are shown at the top and occipital-parietal sensors at the bottom.

**Figure 7** illustrates a sensor-level view of MEG auditory evoked waveforms along with left- and right-hemisphere “M50” magnetic field instantaneous topographic overlays for a 14-month-old participant undergoing stimulation with a 500 Hz sinusoidal tone. Clear bilateral evoked responses are resolved, along with dipolar magnetic field topographies amenable to single equivalent current dipole modeling. Note that the lateral temporal sensors exhibit greater evoked response amplitudes than frontal or occipital sensors, indicating the spatial discrimination of the device.

**FIGURE 8:**
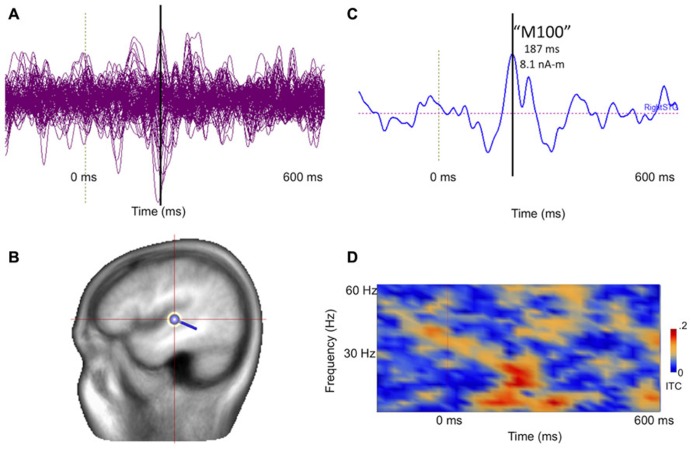
**(A)** Collapsed (“butterfly”) plot of MEG auditory evoked sensor waveforms for a 14-month-old participant experiencing stimulation with a 1000 Hz tone, showing data from all MEG channels with stimulus locked averaging from -200 to 600 ms with respect to tone onset (filtered 3–40 Hz). **(B)** A dipolar regional source is shown for the right superior temporal gyrus in a standard head model. **(C)** Source time course of the right superior temporal gyrus waveform, indicating a clear deflection at approximately 180 ms (vertical line). **(D)** Time-frequency transformation of the single–trial data allows estimation of inter-trial coherence (ITC), or phase-locking, for the source (time on x axis and frequency on y axis). Bright red regions post-stimulus indicate increased superior temporal gyrus phase-locking to the 1000 Hz tone, particularly at lower frequencies.

**Figure 8** illustrates post-processing of the averaged evoked sensor-level response for the 14-month-old participant, showing source space estimation of the time course of the right superior temporal gyrus auditory response, revealing resolvable component events, characterizable in terms of amplitude and latency. **Figure 8D** extends the analysis to the spectrotemporal domain, allowing resolution of stimulus-related inter-trial coherence in lower (<20 Hz) domains.

**FIGURE 9:**
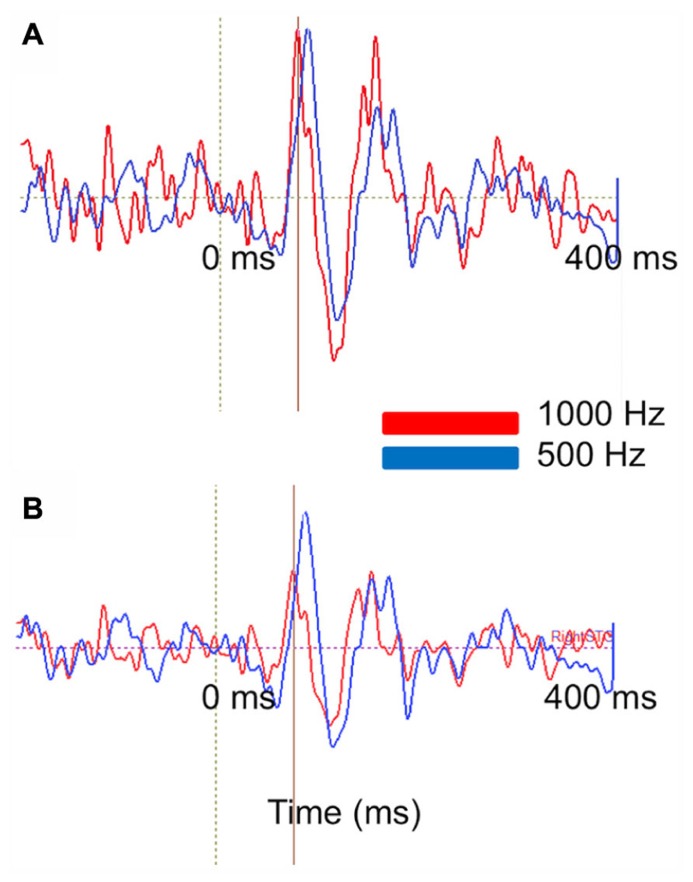
**Stimulus attribute (frequency) encoding is reflected at both the sensor and source levels**. **(A)** PCA on MEG auditory evoked sensor waveforms comparing a 1000 Hz (red) and 500 Hz (blue) auditory response in an 18-month-old-subject. Waveforms are shown from -200 to 400 ms. **(B)** Right superior temporal gyrus source waveforms in the same subject. Time courses are shown from -200 to 400 ms. At sensor and source level a slight latency shift is resolvable (with the higher frequency stimulus eliciting a shorter latency response).

**Figure 9** represents the ability of auditory evoked recordings to assess neuronal encoding of stimulus features such as frequency in young children. In this example, an 18-month-old participant underwent stimulation with 500 and 1000 Hz sinusoidal tones. Sensor-level waveforms were averaged and decomposed using PCA, and the PCA waveforms for each stimulus overlaid to illustrate a stimulus-frequency-dependent latency shift of the M50 evoked response (analogous to that described by Roberts and Poeppel, 1996). Sensor-level findings are recapitulated in source space using regional source modeling, with a similar auditory latency shift observed.

Middle latency responses (analogous to the M50 described in older children and adults) also appear to exhibit a developmental time course. The preliminary data examined in this study were consistent with a maturational shortening of the “M50” latency from 180 ms at 14 months through 156 ms at 18 months to 147 ms at 48 months (500 Hz tone stimulation) as described by Paetau et al. (1995) and Roberts et al. (2013). Of note, data from the youngest participant was acquired during sleep. Sleep may additionally modulate (prolong) evoked response latency.

## DISCUSSION

Present findings show that the Artemis 123 provides high-quality recordings from individuals aged 14 to 48 months. In particular, examination of Artemis 123 auditory data showed generalizability, with auditory responses observed in all three participants. The auditory MEG measures were also found to be manipulable (**Figure 9**), exhibiting sensitivity to tone frequency. Furthermore, there appeared to be a predictable sensitivity of evoked response components to development, with latencies decreasing with age. Given the atypical developmental trajectory of M50 latency in disorders such as ASD (e.g., Roberts et al., 2013), this offers exciting potential for assessment of auditory system development as well as early detection of atypical auditory processing. Examination of resting-state rhythms also showed characteristic resting-state oscillatory activity (posterior resting-state alpha, heartbeat activity), indicating that the Artemis 123 provides quality recordings of resting-state rhythms in young children, with potential for analyses of resting-state functional networks and connectivity. Future work will involve examining the feasibility of obtaining somatosensory and visual recordings in similar-age children as well as obtaining recordings from infants. Finally, phantom data showed that dipole sources could be localized with an error less than 0.5 cm.

Given that in many studies it will not be possible to obtain magnetic resonance imaging (MRI) data from infants and young children, for some studies it will be necessary to apply techniques (some already developed) to align MEG data to template MRIs. As an example, this could accomplished via affine point based registration techniques to align individualized headshape models (derived from head-surface points) to the scalp of age-matched MRI templates. The transformed age-matched MRI template could then be used for visualization and additional head modeling required for source localization. Once developed, in future studies it will also be possible to use the HPI data to determine the location of the child’s head with respect to the MEG sensors and then co-register the MEG and MRI data for source localization. Unlike EEG electrical fields, as MEG magnetic fields are insensitive to changes in conductivity profiles (e.g., between tissue, CSF, skull, skin) a single-shell head model can be used for source localization (Lewine and Orrison, 1995).

There are several unresolved technical challenges. First, given that it is difficult to obtain recordings in young children and infants over an extended period of time, developing paradigms that quickly provide multiple measures is of interest. For example, a paradigm that simultaneously presents auditory, visual, and somatosensory stimuli would allow assessment of the integrity of multiple primary sensory areas in less than five minutes. Second, for longer paradigms and tasks, it will be important to develop procedures to correct for head movement. With the ability to collect MEG data with the HPI coils active throughout the recording, at a minimum this could involve using the HPI information to remove MEG data where the participant’s head moves beyond a threshold. Given the acquisition of continuous HPI data, however, procedures can be developed to compensate for head motion (Stolk et al., 2013). Finally, in some infants (as exemplified in the youngest participant described in this manuscript) it might be necessary to obtain data during sleep (i.e., recordings during naptime or in the evening). For such studies it will be important to determine the effect of sleep on primary sensory (as well as non-task “resting”) activity, motivating detailed comparative studies in awake and sleeping states.

As previously noted, examination of the continuous recordings showed that the Artemis 123 provided quality recordings of resting brain activity as well as common non-brain artifacts (**Figure 6**). Using adult MEG systems, clinical MEG recordings are obtained in individuals with epilepsy to localize the generator(s) of epileptiform activity and also in individuals with lesions to localize eloquent cortex (Roberts et al., 1995, 2000; Roberts and Rowley, 1997; Gaetz et al., 2009). Although clinical MEG studies are performed in infants, a limitation of these studies is that in very young children the MEG sensors are far from the infant’s brain. Clinical MEG infant and young child studies using the Artemis 123 would provide measures of epileptiform activity with significantly higher sensitivity than adult MEG system, thus potentially providing more accurate estimates of the location of brain pathology in clinical infant groups. It remains to be established whether this sensitivity translates straightforwardly to increased signal to noise ratio, given the expected increase in sensitivity to other coincident brain activity.

Although present findings show that quality recordings can be obtained in infants and young children, there are several limitations to consider. First, although the helmet was sized so that sensors can be placed as close as possible to a typical three-year-old’s head, examination of normative head circumference charts shows that two-year-olds with head circumferences above the 90th percentile and three-year-olds with head circumferences above the 95th percentile will not fit into the helmet (see normative charts in Roche et al., 1987; given slightly smaller head circumferences in females than males, more two- and three-year-old girls than boys will fit into the helmet). As such, although the Artemis 123 will accommodate the majority of children three and under, many children four years and older will not fit into the Artemis123 helmet, thus placing limits on the age-range that can be examined in Artemis 123 studies. Although a limitation, reducing helmet size was a major consideration in achieving improved sensitivity to infant and young child brain activity, and with our ongoing interest in early signs of ASD as well as language acquisition during the 12–30 month period, the Artemis 123 helmet size was selected to be optimal for this period of development.

For MEG studies examining brain function in children across a broad age-range, it would be possible to obtain data in the youngest children using the Artemis 123 and in older children using an adult MEG system. Although such studies are clearly of interest, in these studies it will be necessary to determine equivalence in the dependent variables of interest between the infant/young child and adult MEG systems. For example, although latency measures are likely similar between infant and adult MEG systems it remains to be determined if the infant MEG system amplitude measures need to be scaled to be comparable to an adult MEG system. Studies examining children (e.g., a four-year-old) in the Artemis 123 and an adult system are needed. In our laboratory, the Artemis 123 is sited next to an adult CTF 275 system (VSM), making such comparisons possible.

Another limitation worth noting is that, at present it is not possible to correct for head motion and thus it is necessary for the participant to remain still throughout the recording. This is, however, a temporary limitation - the Artemis 123 provides the ability to continuously record head movement via the head coils and, as such, algorithms to correct for head motion can be developed. Finally, although the Artemis 123 has reference channels, these reference channels were not used to remove external noise in the present human data recordings [in the Methods it was shown that a spatial filter can be used to remove external noise from empty room data (e.g., train and 60 Hz power-line noise)]. Although noise-cancellation procedures using the reference channels will be developed, it is of note that quality human recordings were obtained without the use of the reference channels.

To conclude, this manuscript describes the design and implementation of a whole-head biomagnetometer optimized for infants and small children. Phantom studies confirm signal detection and source localization ability. Preliminary infant studies demonstrate recordings at rest and during stimulus presentation and illustrate the generalizability of recordings. Furthermore, auditory evoked response component latencies illustrate sensitivity to stimulus features and potentially representing the neural processes underlying feature encoding and representation in the brain. Finally, latencies appear to mature (shorten) with increasing age, potentially indexing development of primary and secondary auditory areas. These latter two attributes (representation and development) have been observed as atypical in certain disease populations (including ASD). The Artemis 123 offers promise as a means of detecting earlier diagnostic signatures in such neurodevelopmental disorders.

## Conflict of Interest Statement

Several of the authors (Mascarenas, Miller, Han, Caffrey, Kincade, and Power) are employees of Tristan Technologies Inc.. Drs Paulson and Pratt have ownership interests in Tristan Technologies Inc. Dr Hirschkoff is a board member and consultant for Tristan Technologies Inc. Dr. Roberts has served as a paid speaker on behalf of Elekta Oy and Siemens Medical Solutions. Additionally Dr. Roberts holds stock options in Prism Medical Imaging in return for consulting services. Other authors have no conflicts of interest to report.
